# Six-year data from three magnetic resonance-guided radiotherapy systems demonstrate long-term stability of linear accelerator quality assurance

**DOI:** 10.1016/j.phro.2026.101091

**Published:** 2026-09-23

**Authors:** Guido C. Hilgers, Simon J. Woodings, Thomas A. Foppen, Jochem W.H. Wolthaus, André W. Minken, Bas W. Raaymakers, Bram van Asselen

**Affiliations:** a Department of Radiotherapy, University Medical Center Utrecht, Utrecht, The Netherlands; b Radiotherapiegroep, Arnhem/Deventer, The Netherlands

**Keywords:** Radiotherapy, Quality assurance, MRI-Linac, Unity

## Abstract

**Background and Purpose:**

Magnetic resonance-guided radiotherapy systems require comprehensive quality assurance (QA) programs to ensure safe and precise treatment delivery. This study evaluates the long-term behavior and variability of linac-related QA results across three clinically operated 1.5 T MRI-linacs at a single institution and assesses compliance with tolerance limits.

**Materials and Methods:**

QA data acquired over a six-year period from three clinically operated 1.5 T MRI-linacs were retrospectively analyzed. A range of linac-related dosimetric and mechanical QA tests was included. Performance envelopes (mean ± 2 SD) were constructed to characterize variability relative to tolerance limits.

**Results:**

For 11 of 13 QA tests, performance envelopes for the associated metrics remained within the applied tolerance limits. For the two remaining tests, envelopes slightly extended beyond these limits. These deviations were not considered clinically relevant, and their underlying causes were understood. No progressive trends of clinical relevance were observed in the linac-specific time series.

**Conclusions:**

Stability of linac-related QA results was demonstrated over six years across three MRI-linacs, supporting reconsideration of the QA program.

## Highlights

• Stability of performance demonstrated across three systems (14 linac-years).
• Performance envelopes remained within applied tolerance limits for 11 of 13 tests.
• Tests have facilitated a better understanding of the system.
• No progressive trends were observed in linac-specific time series.
• Findings support optimization of tolerance limits and test frequencies.

## Graphical abstract

**Figure:**
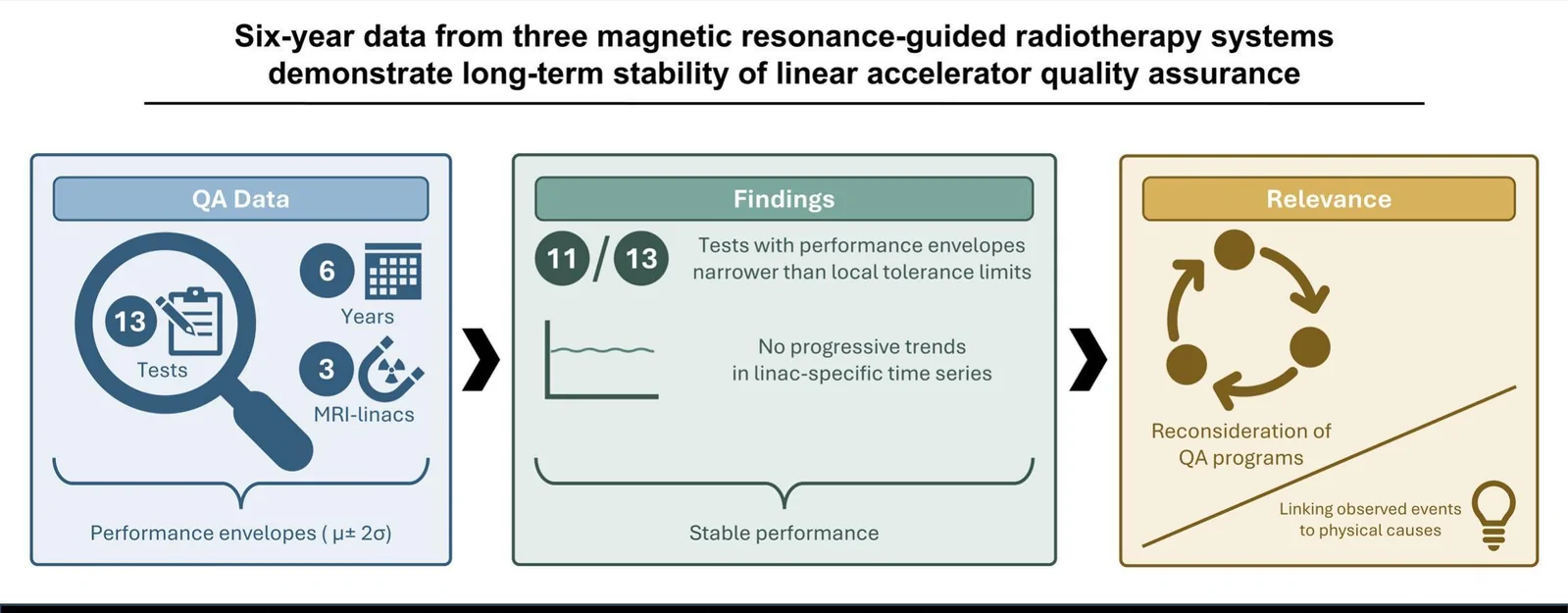

## 1 Introduction

Magnetic resonance-guided radiotherapy (MRgRT) systems [1] integrate a linear accelerator (linac) with a magnetic resonance-imaging (MRI) scanner, enabling real-time soft-tissue visualization during treatment. As with all radiotherapy devices, these systems require a comprehensive quality assurance (QA) program to ensure reliable and reproducible operation, so that safe, accurate, and precise treatments can be delivered [2].

For emerging technologies such as MRgRT systems, many QA tolerance limits and test frequencies are defined during early clinical adoption, while their suitability over prolonged clinical operation remains to be demonstrated. Winter et al. [3], Subashi et al. [4], and Xiao et al. [5] independently demonstrated the stability of linac-related QA results for a 1.5 T MRI-linac during the first year of clinical operation, each using a single machine.

Extending these findings, the present study evaluates the long-term stability of linac-related QA results over a six-year observation period across three 1.5 T MRI-linacs. Their temporal behavior and variability are examined, and compliance with applied tolerance limits is assessed, thereby contributing to the ongoing development of QA programs. Longitudinal assessment of MRI-related QA results was beyond the scope of this study.

## 2 Materials and methods

Data from three clinical Unity 1.5 T MRI-linacs (Elekta, Stockholm, Sweden) were retrospectively analyzed. Measurements were acquired as part of routine QA between January 1, 2019, and December 31, 2024. The three systems (U01, U02, and U03) were located at University Medical Center Utrecht, the Netherlands.

The Unity system [6], [7], [8] is equipped with a 7 MV flattening-filter-free (FFF) photon beam with a nominal dose rate of 425 MU·min^−1^. Beam shaping is provided by two cross-plane jaws and an in-plane 80-leaf-pair multi-leaf collimator (MLC). At the isocenter, located 143.5 cm from the radiation source, the maximum field size is 57 cm (cross-plane) × 22 cm (in-plane). The linac, including the beam limiting device (BLD), can be retracted from the ring gantry structure for maintenance using a swing-in, swing-out (SISO) winch system. An on-board megavoltage imager (MVI) with a field-of-view (FOV) of 21.0 cm × 8.5 cm is used for machine setup and BLD calibration. The system does not rely on an external laser positioning system, and couch motion is limited to translation along the patient longitudinal (head–foot) direction. Clinical treatments are currently delivered using step-and-shoot intensity modulated radiotherapy (IMRT).

All three MRI-linacs underwent acceptance testing according to the same procedure [9]. A uniform QA program was subsequently applied. While broadly consistent with later-developed Elekta MRI-linac consortium guidelines [8], this program incorporated selected tests employing stricter tolerances or modified test frequencies informed by clinical experience. Table 1 summarizes the tests evaluated in this study. Further details are provided below.

**Table 1:** Tests, equipment, frequencies, local tolerance limits, and performance envelopes (mean ± 2 standard deviations). Frequency: D = daily; W = weekly; 2W = biweekly; M = monthly; Q = quarterly; SO = after swinging out the linac from the gantry ring structure. Other abbreviations: MU = monitor unit; MVI = megavoltage imager; pp = percentage point.

**Test**	**Equipment**	**Frequency**^a^ **[Observations]**^b^	**Local tolerance limit**	**Performance envelope**^c^	
***Dosimetric:***					
Output constancy	On-board MVI	D [712, 1377, 697]	±3% from baseline	0.1 ± 1.5%	
Output check	Ionization chamber Water-equivalent plastic	W (2019–2022) 2W (2023–2024) [206, 279, 119]	±1.5%	−0.1 ± 1.1%	
Dose rate	–	W (2019–2022) 2W (2023–2024) [161, 234, 115]	425 ± 25 MU/min	423 ± 14 MU/min	
Backup monitor	–	W (2019–2022) 2W (2023–2024) [161, 232, 116]	100.0 ± 2.0 MU	99.9 ± 0.4 MU	
Dose linearity^d^	Ionization chamber Water-equivalent plastic	W (2019–2022) 2W (2023–2024) [157, 205, 107]	15 MU: ≤1% 5 MU: ≤2%	15 MU: 0.3 ± 0.6% 5 MU: 0.4 ± 0.7%	
X-ray beam quality	Ionization chamber Water-equivalent plastic	W (2019–2022) 2W (2023–2024) [163, 233, 111]	±0.005 from baseline^e^	0.000 ± 0.003	
Beam profile	2D linear detector array	M	*CAX deviation*^f^*:*	x:	1.7 ± 1.3 mm
		[41, 63, 29]	Monitoring only	y:	0.0 ± 1.6 mm
			*Beam size*^g^*:*	x:	0.1 ± 0.5 mm
			±2 mm from nominal size	y:	−0.3 ± 0.8 mm
			*Flatness*^h^*:*	x:	0.1 ± 1.8 pp
			±2 pp from baseline	y:	−0.1 ± 0.8 pp
			*Symmetry*^j^*:*	x:	0.0 ± 0.7 pp
			±3 pp from baseline	y:	0.0 ± 0.9 pp
Liquid helium level	–	D (2019–2023) M (2024) [154, 195, 96]	Monitoring only	U01: U02: U03:	∼5.5 pp ∼1.0 pp ∼1.0 pp
***Mechanical:***					
Focal spot alignment	Radiochromic film	Q + SO [48, 26, 21]	±1 mm	x:	−0.3 ± 0.3 mm
Opposing beam alignment	Radiochromic film	Q + SO	±1 mm	x:	−0.3 ± 0.2 mm
		[32, 48, 22]		y:	−0.1 ± 0.1 mm
Isocenter radius	Radiochromic film	SO [24, 32, 12]	≤ 0.5 mm		0.4 ± 0.3 mm
Leaf position accuracy	On-board MVI	Q + SO [44, 57, 35]	±0.25 mm	y:	0.0 ± 0.1 mm
MR-to-MV alignment^k^	MR MV phantom	M	*Translations:*	x:	0.0 ± 0.1 mm
		[39, 70, 50]	±0.5 mm from baseline	y:	0.0 ± 0.1 mm
				z:	0.1 ± 0.1 mm
			*Rotations:*	ψ:	0.0 ± 0.1°
			±0.3°	φ:	0.0 ± 0.2°
				θ:	0.1 ± 0.3°
			*Mean absolute rotation:*		0.1 ± 0.0°
			≤ 0.2°		

Notes

a Frequencies for several dosimetric tests were reduced from weekly to biweekly from 2023 onward, and liquid helium level checks from weekly to monthly from 2024 onward, based on accumulated clinical experience.

b Numbers in square brackets give the numbers for linac U01, U02, and U03, respectively. Observation counts differed between linacs because of differences in commissioning dates, periods of reduced system availability, and incomplete QA histories associated with the retrospective nature of the dataset.

c Except for liquid helium level, for which the total decrease is reported (see Fig. 4 for details).

d The reduced monitor unit setting was decreased from 15 MU to 5 MU in April 2022, and the local tolerance was consequently widened from ≤1% to ≤2%. Observation counts were N = [94, 106, 18] at 15 MU and N = [63, 99, 89] at 5 MU.

e ${\mathrm{TPR}}_{20,10}$ measured in water-equivalent plastic, corresponding to a relative deviation of ±0.7% in a full-scatter water tank.

f Distance from the midpoint of penumbra inflection points to the physical center of the array.

g Distance between the two penumbra inflection points.

h Mean of the two ratios (dose at 80% of half-beam width) ÷ (dose at CAX).

j According to IEC 60976.

k According to IEC 61217; see Fig. 1a in Roberts et al. [8]; ψ, φ, and θ denote rotations about x-, y-, and z-axes, respectively. Each MRI-linac is supplied with a phantom provided by the manufacturer.

Long-term behavior and variability of QA results were characterized using aggregated data. For each metric, a mean weighted by the number of observations per linac was calculated. Between-linac and within-linac standard deviations (SDs) were calculated separately and combined into a single overall SD. A performance envelope, defined as the mean ± 2 SD, was then assessed against the local tolerance limits.

All directions are defined in the IEC 61217 coordinate system (see Fig. 1a in [8]), with rotations ψ, ϕ, and θ about the x-, y-, and z-axes, respectively.

### 2.1 Dosimetric tests

Daily output constancy was assessed using the MVI and a 100 MU, 7 cm × 7 cm beam at a 90° gantry angle. Along with the acquired image, a pixel factor was reported that is inversely proportional to the delivered dose [10]. The reciprocal of the pixel factor was calculated and compared with the baseline value. Deviations were expressed as a percentage difference.

Output checks were performed using a PTW 30013 Farmer-type waterproof ionization chamber placed in a rigidly positioned, 18.7 cm-thick RW3 water-equivalent plastic phantom (PTW, Freiburg, Germany). Measurements were acquired at isocenter, at a geometric depth of 10 cm, using a 100 MU, 10 cm × 10 cm beam at a 90° gantry angle. Water droplets were added to the cavity to eliminate air gaps [11]. The chamber reading was converted to dose-to-water using the formalism of van Asselen et al. [12] together with an experimentally determined correction factor for the RW3 setup, and compared with the reference dose. Deviations were expressed as percentage differences.

Dose rate constancy and backup monitor chamber constancy were manually recorded based on linac-reported readings at the completion of each output check. Deviations were expressed as absolute differences from the corresponding reference values.

Dose linearity was assessed by repeating the output check at reduced monitor unit settings. The ratio of the chamber reading to the delivered monitor units was calculated and compared with the corresponding reading-to-MU ratio from the output check [13]. Deviations were expressed as percentage differences.

X-ray beam quality was assessed using the tissue-phantom ratio ${\mathrm{TPR}}_{20,10}$ in RW3. An additional 10 cm of water-equivalent plastic was placed upstream of the detector, increasing the geometric depth to 20 cm while maintaining isocenter positioning. A beam identical to that used for the output check was delivered, and the ratio of the uncorrected chamber reading to the uncorrected reading from the output check was calculated. Deviations were expressed as absolute differences from the system-specific baseline.

Beam profiles were acquired by irradiating a STARCHECK maxi MR linear detector array (PTW, Freiburg, Germany) [14], [15] with a 200 MU, 36 cm × 22 cm beam at a 0° gantry angle. The array was positioned at isocenter using the system-specific QA Alignment Platform [8]. Build-up was provided by a polymethyl methacrylate slab corresponding to a water-equivalent thickness of 2.5 cm. Data analysis was performed using BeamAdjust v2.3.3.8. Deviations were expressed in mm or percentage points (pp) from the system-specific baselines.

The liquid helium level inside the cryostat was manually recorded using the manufacturer-provided “Display Helium Level” application available from the MRI console because variations may affect beam attenuation.

### 2.2 Mechanical tests

Focal spot alignment relative to the gantry axis (isocenter) was assessed using Gafchromic EBT3 radiotherapy film (Ashland, Bridgewater, NJ). The film was positioned in the coronal plane at isocenter and sandwiched between RW3 slabs, with additional copper plates included to reduce the influence of the 1.5 T magnetic field on dose deposition [16]. The film was irradiated with two opposing MLC-only-defined half-beams (20 cm × 5 cm) delivered at gantry angles of 0° and 180° (see Fig. 1a). Under ideal alignment, the resulting images form a centered 20 cm × 10 cm rectangle (see also Fig. 4 in [9]). Alignment was assessed by determining the x-offset of the 180° image relative to the 0° image. The magnitude of the alignment deviation was defined as half of this offset and determined using in-house MATLAB software (The MathWorks, Natick, MA).

**Fig. 1:**
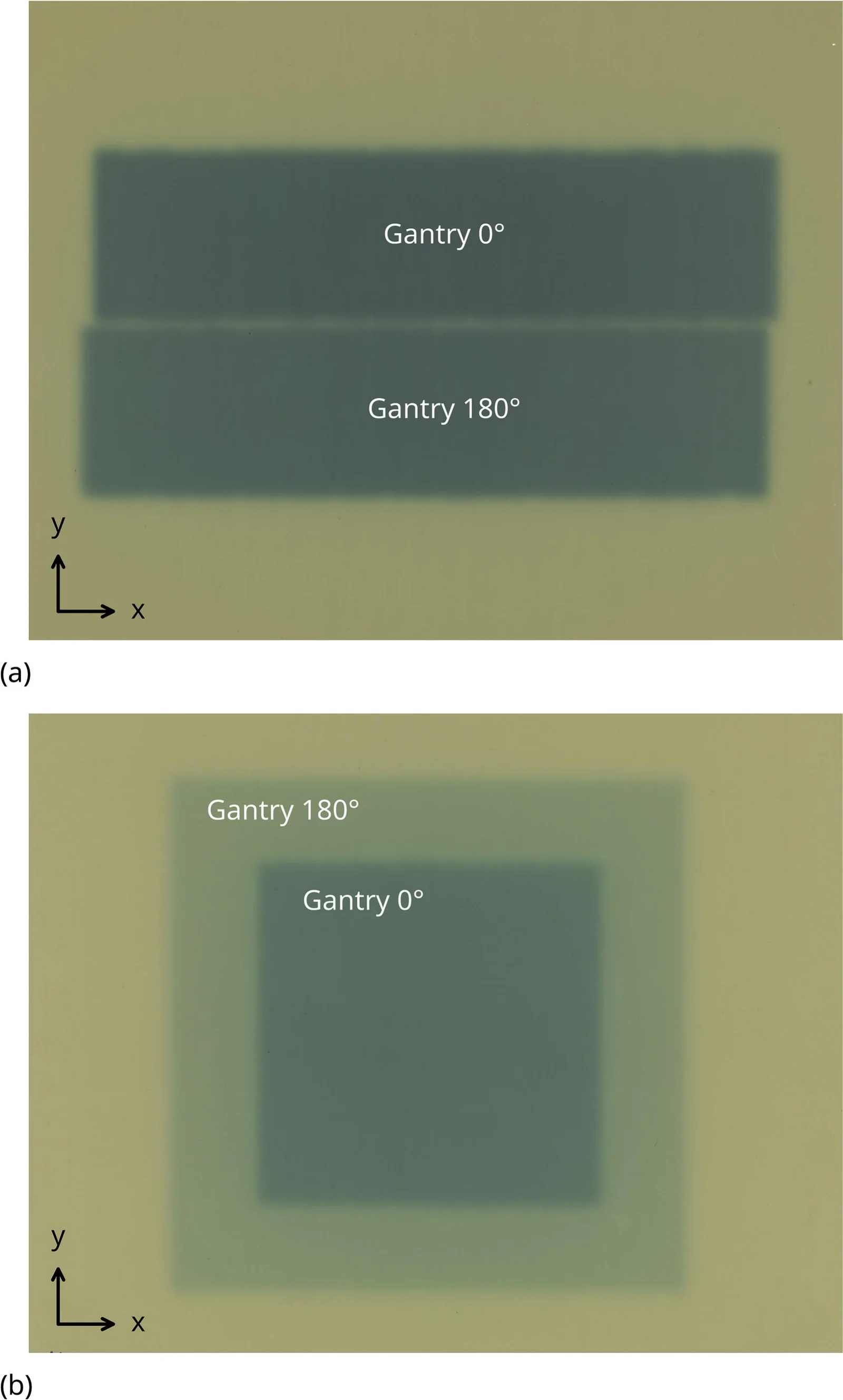
Radiochromic films after exposure for focal spot alignment (a) and opposing beam alignment (b). In focal spot alignment, two half-beams defined by leaf sides and tips are delivered at opposing gantry angles. Ideally, these align in the IEC 61217 x-direction. In opposing beam alignment, two beams defined by jaws and leaf tips are delivered at opposing gantry angles. Ideally, their centers coincide.

Opposing beam alignment was assessed using the same experimental setup. The film was irradiated with a centered 10 cm × 10 cm beam delivered at a 0° and a centered 15 cm × 15 cm beam delivered at a 180° gantry angle (see Fig. 1b). In contrast to the focal spot alignment test, beam shaping was performed using the jaws and the MLC. Alignment was assessed by determining the x- and y-offsets of the 180° image relative to the 0° image. The alignment deviations were defined as half of the corresponding offsets and determined using in-house MATLAB software.

Isocenter accuracy during gantry rotation was assessed using a star-shot measurement on Gafchromic EBT3 radiotherapy film. The film was positioned in the transverse plane at isocenter between copper rings to mitigate the effect of the magnetic field on dose deposition [16]. It was irradiated with twelve 1.4 cm-wide MLC-defined beams delivered at gantry angles of 195°–345° and 0°–150°, in equidistant 30° increments. The isocenter radius was determined using in-house MATLAB software [17].

Leaf position accuracy was assessed using an MVI-based strip test [18]. Five MLC-defined strips of 2 cm width separated by 1 mm gaps were delivered, with the jaws retracted. This produced an image containing four abutments per leaf pair, yielding 120 abutments for the central 30 leaf pairs; the other leaf pairs were outside the MVI's FOV. For each abutment, the displacement of the midpoint between opposing leaf ends was calculated as the absolute positioning error (APE) using in-house MATLAB software. The median APE across all 120 abutments was reported.

Alignment between the magnetic resonance (MR) and megavoltage (MV) coordinate systems was assessed according to the manufacturer's test procedure [8]. This test verifies that the coordinate transformation between the MRI and linac subsystems remains consistent with the commissioned state, a necessary condition for accurate and precise treatment delivery.

## 3 Results

Table 1 presents the performance envelopes observed over a cumulative period of approximately 14 linac-years. These remained within the applied tolerance limits for nearly all QA metrics. Only the isocenter radius and MR-to-MV rotational alignment about the z-axis (θ) showed envelopes that slightly extended beyond these limits (see Fig. 2). For many metrics, performance envelopes were substantially narrower than the corresponding tolerance limits. Examples include output constancy (0.1 ± 1.5% versus ±3.0%), beam profile symmetry (0.0 ± 0.7 and 0.0 ± 0.9 pp versus ±3 pp), and focal spot alignment (−0.3 ± 0.3 mm versus ±1 mm).

**Fig. 2:**
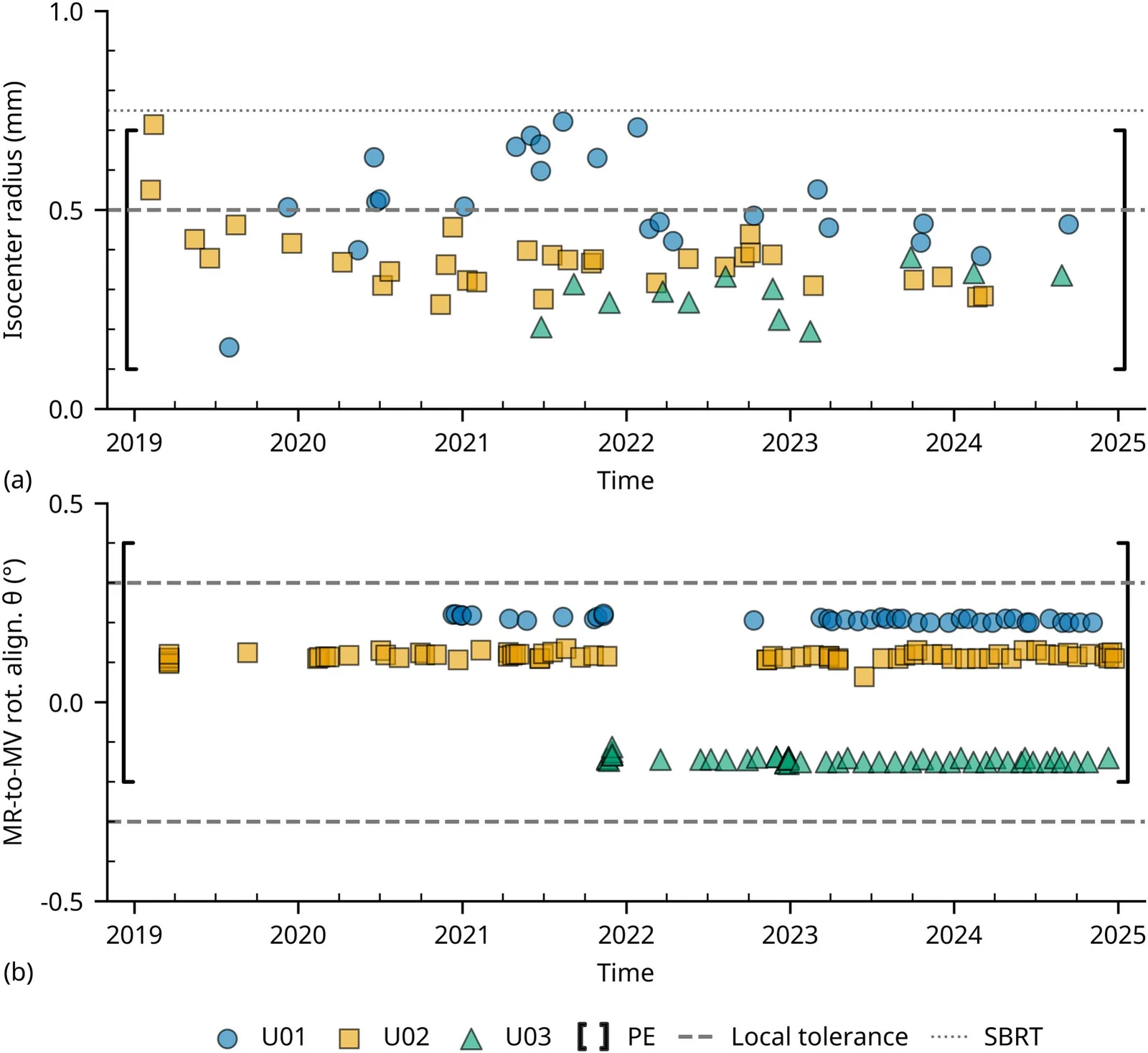
Individual measurements for metrics whose performance envelope (PE) slightly extended beyond the local tolerance: isocenter radius (a) and MR-to-MV rotational alignment (rot. align.) about the z-axis (b). The dotted line indicates the national stereotactic body radiotherapy (SBRT) recommendation [20]. In (b), gaps in the U01 and U02 series indicate periods for which measurement data were unavailable.

Two QA metrics exhibited distinct temporal patterns. X-ray beam quality showed a recurring pattern with occasional step changes, most evident for linac U02, while remaining within the tolerance band (see Fig. 3). Liquid helium levels showed step changes and, for linac U03, a gradual decrease over time (see Fig. 4).

**Fig. 3:**
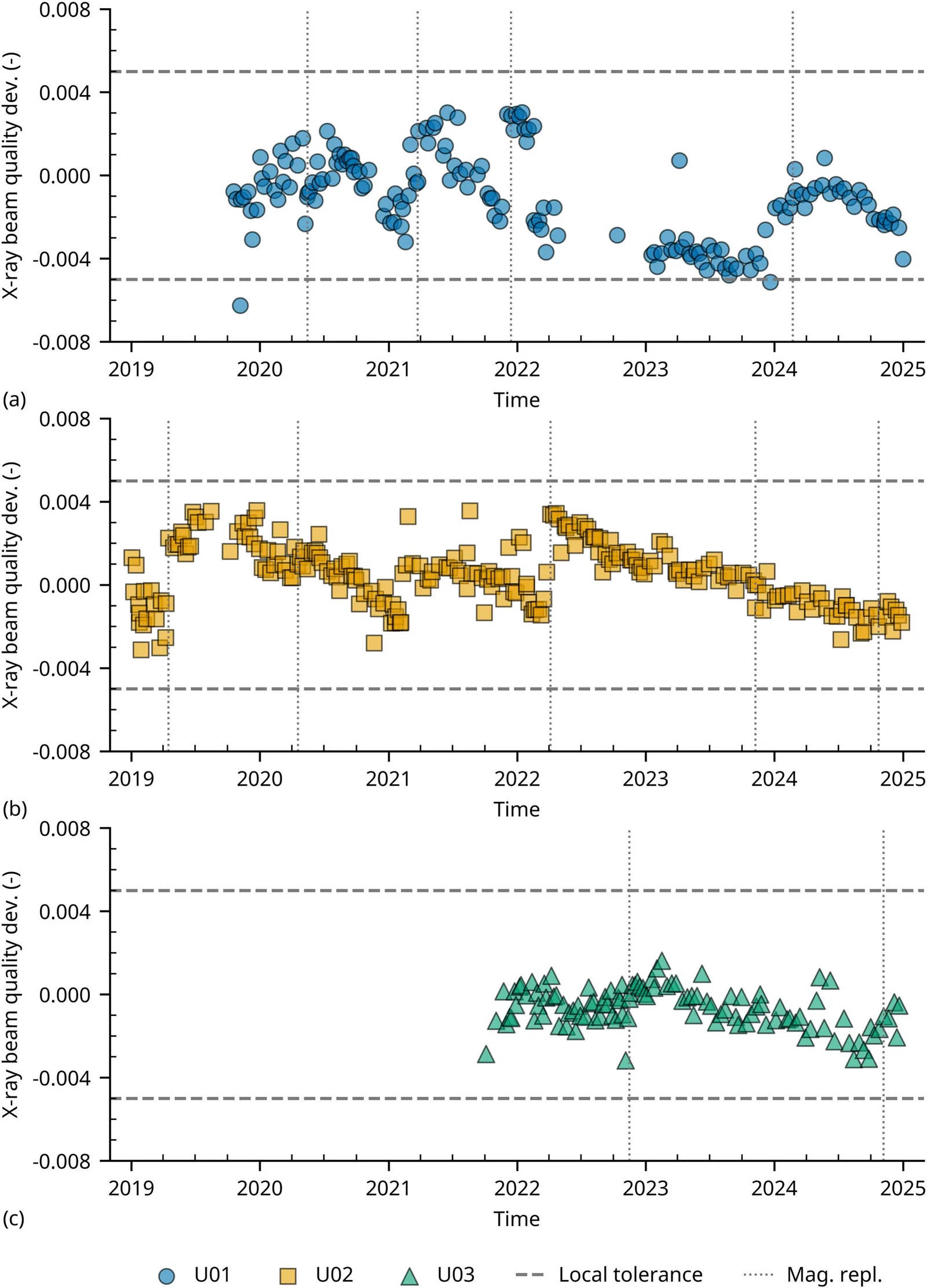
Beam quality deviations (dev.) for linacs U01 (a), U02 (b), and U03 (c). Magnetron replacements (mag. repl.) are indicated with vertical dotted lines. The local tolerance limits correspond to a relative tissue-phantom ratio ${\mathrm{TPR}}_{20,10}$ deviation in water of ±0.7%.

**Fig. 4:**
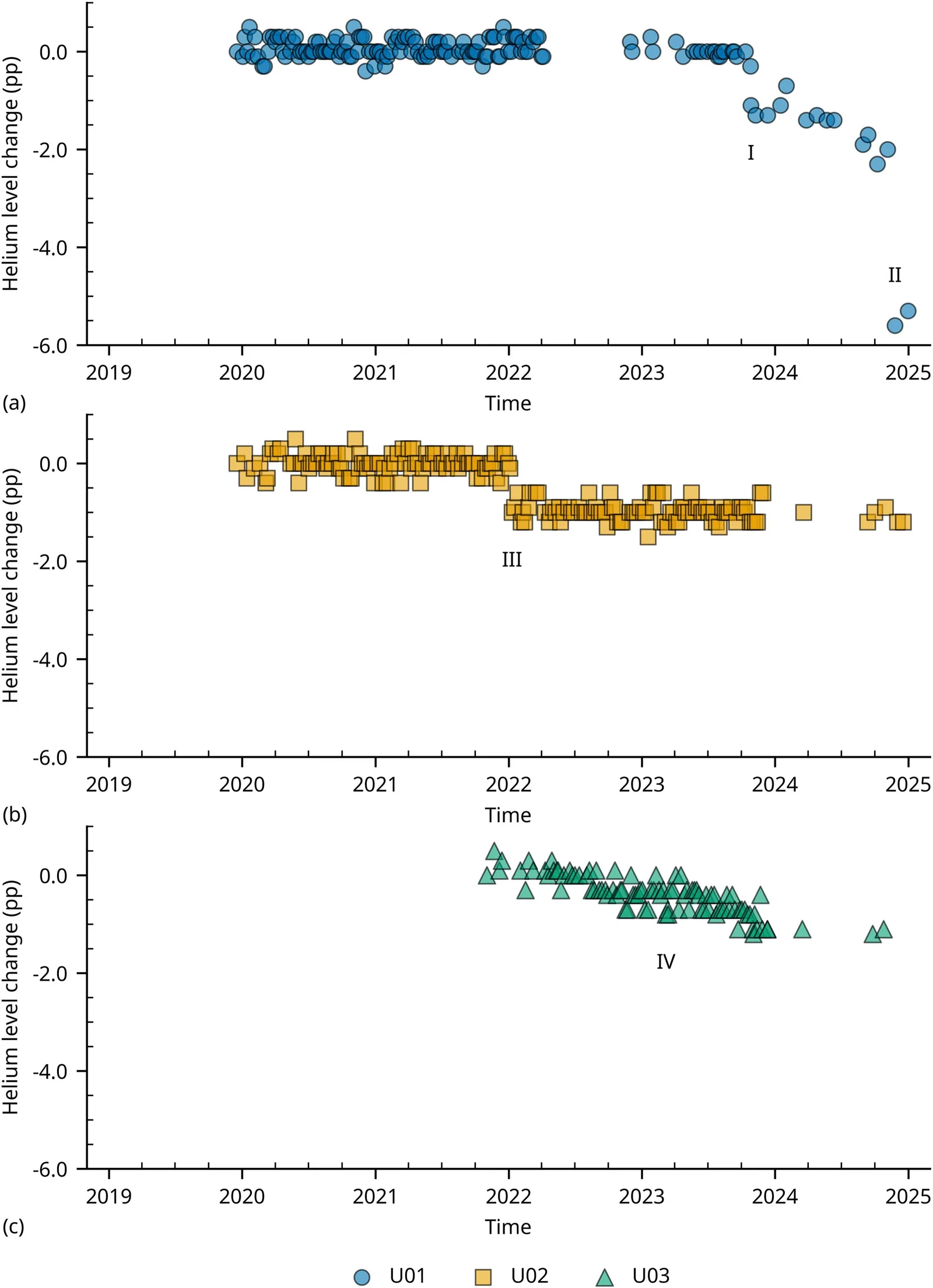
Liquid helium level for linacs U01 (a), U02 (b), and U03 (c), relative to the respective baseline levels (U01: 97.7%, U02: 99.2%, U03: 90.9%) and expressed in percentage points (pp). Liquid helium levels remained well above the 30% threshold for manufacturer intervention related to MRI system operation. In (a), the gap indicates a period for which measurement data were unavailable. Annotations: I, III = after cold-head (helium re-condenser) maintenance; II = after unavoidable ramp-down and ramp-up of the magnetic field due to a gradient coil replacement; IV = small leak until 2024.

Although the performance envelopes for both focal spot alignment and opposing beam alignment were well within tolerance, consistent directional shifts were observed. The mean focal spot alignment was −0.3 mm, indicating a consistent shift in −x direction (see Fig. 5a). Only one measurement demonstrated a clear shift in the +x direction, which was traced to an alignment issue. The mean opposing beam alignment along the x-axis (see Fig. 5b) matched the mean focal spot alignment in both magnitude and direction. One measurement exhibited a pronounced deviation, which was likewise attributed to an alignment issue. In contrast, the mean opposing beam alignment along the y-axis was close to zero (see Fig. 5c).

**Fig. 5:**
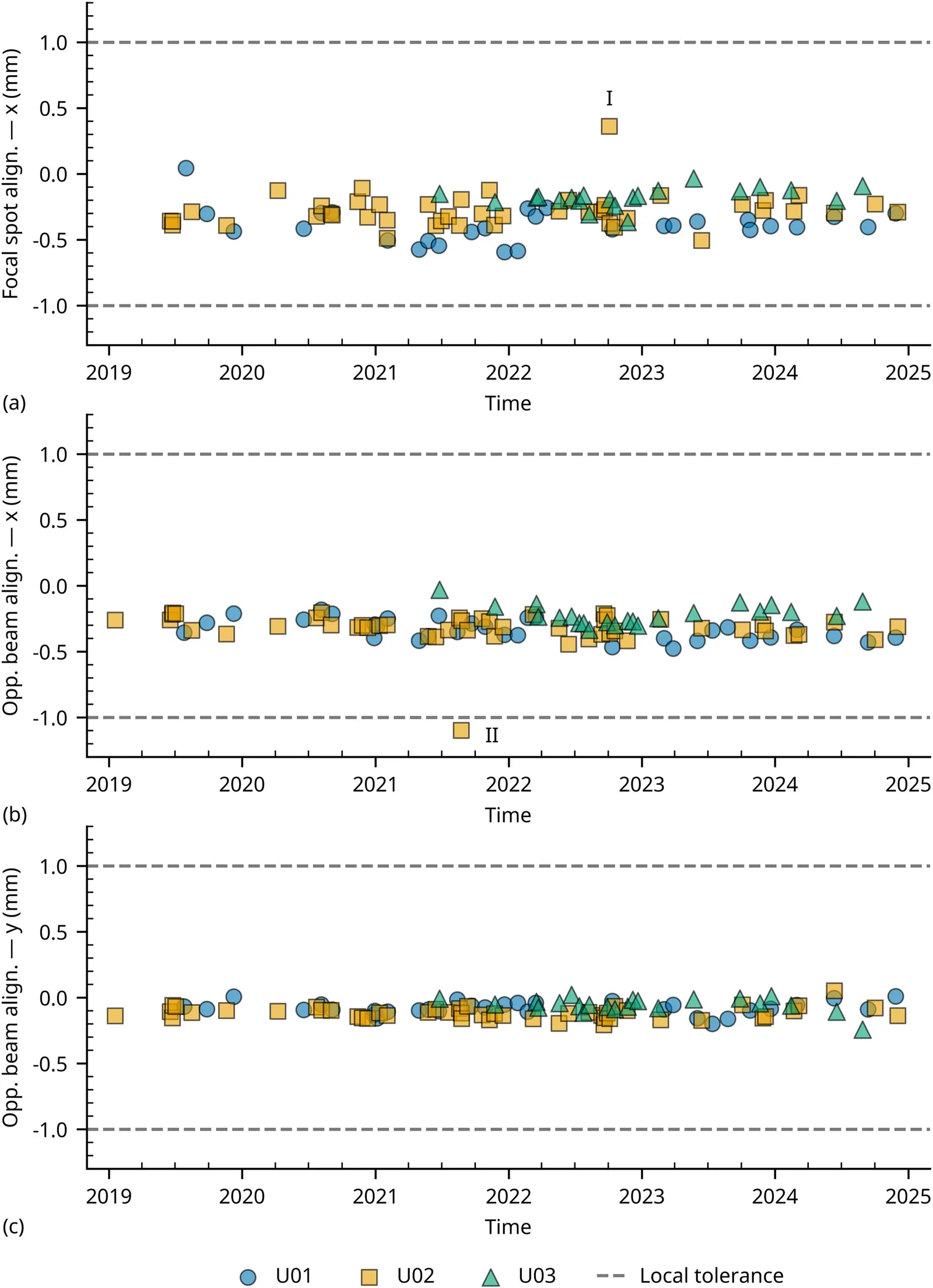
Focal spot alignment (focal spot align.) along the IEC 61217 x-axis (a), and opposing beam alignment (opp. beam align.) along the IEC 61217 x- and y-axes (b, c). Annotations: I = the linac was not fully returned to its correct position after maintenance; II = the beam limiting device had been calibrated before the linac was fully restored to its correct position after maintenance.

## 4 Discussion

This study evaluated the long-term behavior of linac-related QA results. Overall, the results indicate stable linac-related QA performance over a cumulative period of approximately 14 linac-years. Across 11 of the 13 tests, performance envelopes for the associated metrics remained within the applied tolerance limits, with two exceptions that slightly extended beyond these limits, as discussed below.

For the isocenter radius, the performance envelope was found to extend beyond the applied tolerance limit of 0.5 mm (see Fig. 2a). This extension was partly influenced by individual out-of-tolerance measurements on linac U01 between May 2021 and January 2022. During this period, the manufacturer reported an isocenter radius within tolerance using a Winston-Lutz-type test [19]. Star-shot films, when applied on an MRI-linac, may overestimate the isocenter radius due to incomplete correction of the Lorentz effect. In addition, the larger values may also relate to beam alignment, as a small shift in focal spot alignment was observed during this period. From a clinical perspective, recent recommendations for stereotactic body radiotherapy (SBRT) by the Netherlands Commission on Radiation Dosimetry [20] state that an isocenter radius below 0.75 mm is preferred. Both the individual measurements and the performance envelope fall within this recommended range.

For MR-to-MV rotational alignment about the z-axis (θ), the performance envelope extends beyond the applied tolerance limits of ±0.3° (see Fig. 2b). This extension is driven by between-linac rather than within-linac variability. The persistent between-linac differences may reflect differences established during commissioning. Although each MRI-linac was initially assessed using its manufacturer-supplied phantom, a single phantom was used for all systems from 2023 onward after validation. Phantom-related effects are therefore unlikely to account for the observed differences. Accordingly, the observed envelope extension is not considered clinically relevant.

Two metrics exhibited distinct temporal patterns, with X-ray beam quality, and thus nominal beam energy, showing a recurring pattern. The timing of pronounced changes appeared to coincide with magnetron replacements (see Fig. 3), and magnetron optimization may also have contributed to the observed pattern. The overall trend is suggestive of a gradual, age-related decrease in magnetron performance. All changes were minor and remained well within the applied tolerance limits.

The liquid‑helium level showed stepwise decreases following cold-head (helium re-condenser) maintenance and gradient-coil replacement (see Fig. 4). Although such stepwise decreases were anticipated by Roberts et al. [8], they have not, to the best of our knowledge, previously been demonstrated in published long-term operational data. For context, Jelen et al. [21] reported that an approximately 8.5% helium top-off resulted in a dose reduction of less than 0.5% in clinical treatment plans, depending on beam arrangement. Under specific conditions, however, variations in liquid helium level can lead to systematic dose deviations approaching 1%, depending on the helium level at commissioning, the gantry angle used for beam output calibration, and whether a relevant helium level threshold has been crossed (see §7.4.1 in [22]).

A systematic directional shift of −0.3 mm along the x-axis was observed for both focal spot alignment and opposing beam alignment. This may arise from a combination of effects. The sign of the shift is compatible with a possible residual Lorentz-force measurement artifact, despite the use of copper to mitigate magnetic-field effects. Very small deformation of the gantry ring structure may also contribute to the observed displacement. Data presented by Hunt et al. [23] suggest an effect of approximately −0.1 mm.

The focal spot alignment test was originally intended to measure the “top” position of the FFF beam. However, the associated uncertainty was found to be too large for reliable interpretation, but beam edge analysis was still possible. Focal spot alignment and opposing beam alignment differ in how the beam is defined along the x-axis. For focal spot alignment, the beam is shaped by MLC leaf sides, whereas for opposing beam alignment the jaws are used. As a result, focal spot alignment is independent of BLD calibration, while opposing beam alignment reflects a combination of focal spot position and BLD calibration effects.

The beam profile measurements using a 2D linear detector array are affected by alignment constraints arising from small residual positional variation of the QA platform supplied with the MRI-linac relative to the treatment couch (∼1–1.5 mm). Alignment is further complicated by the size and weight of both the platform and array. This is reflected in the width of the performance envelopes for the CAX deviation. Residual positioning variation may cause measured profiles to appear shifted relative to the commissioning baseline, without indicating a true beam shift. In this study, beam size, flatness, and symmetry were unaffected by this variation, as these metrics were referenced to the midpoint of the penumbra inflection points rather than the array center. As expected, a non-zero central value in the +x direction was observed due to magnetic field-induced effects [8].

Performance envelopes provide a concise summary of long-term QA behavior relative to tolerance limits. Their width reflects both between-linac and within-linac variability, indicating whether limits are well matched, too tight, or too wide. Central values, however, require more careful interpretation. For metrics expressed as relative values, they are expected to be near zero. For metrics expressed as absolute values, they reflect the central tendency of the aggregated data and should not be used to re-center tolerance limits (illustrated by the MR-to-MV rotational alignment about the z-axis). Performance envelopes therefore indicate whether QA metrics remain within tolerance limits at the aggregated level, but do not characterize linac-specific drift or trends. These were assessed separately by examining the longitudinal time series for each linac. Together, compliance with tolerance limits at the aggregated level and the absence of observed progressive deviations in linac-specific time series demonstrate stable long-term QA results under routine clinical operation.

While tolerance limits may be adjusted, measurement frequencies may also be revisited. The present data do not, however, permit identification of minimum safe testing frequencies for individual QA procedures. Risk-based approaches [24] such as the Dutch QA2030 initiative [25] may provide a framework for establishing such testing frequencies. Several dosimetric tests evaluated in this study had already undergone a reduction in frequency (see Table 1). Further reductions may be considered, subject to local assessment. Where more frequent monitoring of linac-specific drift is necessary, daily automated MV imager-based checks are available for beam output and X-ray beam quality [26], [27], [28]. In addition, the long-term stability, demonstrated using routine QA tests, may indicate a reduced need for water tank measurements in clinical operation under standard conditions.

Recently introduced options, such as motion management [29], [30], and future delivery techniques, including volumetric modulated arc therapy [31], [32], may require additional linac-related tests not covered by the present QA program. The long-term stability of these tests across extended periods and multiple MRI-linacs remains to be established. Longitudinal assessment of MRI-related QA results was beyond the scope of the present study and has been addressed previously by Wetscherek et al. [33] for up to two years across eight MRI-linacs.

Potential residual magnetic field effects in film-based measurements using copper warrant further study. A systematic comparison of isocenter radii derived from star-shot films and manufacturer measurements may help to better characterize the differences between these methods. Furthermore, refinement of QA platform design may facilitate accurate and reproducible positioning of 2D linear detector arrays during beam profile measurements.

In conclusion, stability of linac-related QA results was demonstrated over six years across three MRI-linacs, extending prior one-year, single-system investigations and supporting reconsideration of the QA program.

## Declaration of generative AI and AI-assisted technologies in the manuscript preparation process

During the preparation of this work the author(s) used Copilot (Microsoft, Redmond, WA) in order to edit text. After using this tool/service, the author(s) reviewed and edited the content as needed and take(s) full responsibility for the content of the publication.

## CRediT authorship contribution statement

**Guido C. Hilgers:** Writing – review & editing, Writing – original draft, Visualization, Methodology, Investigation, Formal analysis, Conceptualization. **Simon J. Woodings:** Writing – review & editing, Supervision, Methodology, Investigation, Conceptualization. **Thomas A. Foppen:** Writing – review & editing, Software, Methodology, Investigation, Conceptualization. **Jochem W.H. Wolthaus:** Software, Conceptualization, Writing – review & editing. **Andre W. Minken:** Writing – review & editing, Supervision, Conceptualization. **Bas W. Raaymakers:** Writing – review & editing, Supervision, Conceptualization. **Bram van Asselen:** Writing – review & editing, Supervision, Conceptualization.

## Declaration of competing interest

The authors declare the following financial interests/personal relationships which may be considered as potential competing interests: University Medical Center Utrecht and Radiotherapiegroep have reference site agreements with Elekta, and University Medical Center Utrecht receives cash and in-kind research support from Elekta.
